# Supplementary material for: Validation of the Standardized Needs Evaluation Questionnaire in Polish Cancer Patients
Source: Cancers (Basel). 2024 Apr 9;16(8):1451. doi: 10.3390/cancers16081451 (PMC11048258; doi:10.3390/cancers16081451)
Supplement: Supplementary file 1 [file cancers-16-01451-s001.zip › cancers-2919324-supplementary/Supplementary material/Suppl. 1.pdf]

Suppl. 1. Polish version of Needs Evaluation Questionnaire

KWESTIONARIUSZ Nr ……..

1. Potrzebuję więcej informacji na temat mojej choroby
   - ☐ Tak
   - ☐ Nie
2. Potrzebuję więcej informacji na temat mojego stanu zdrowia w przyszłości
   - ☐ Tak
   - ☐ Nie
3. Potrzebuję więcej informacji dotyczących wykonywanych badań
   - ☐ Tak
   - ☐ Nie
4. Potrzebuję więcej informacji na temat mojego leczenia
   - ☐ Tak
   - ☐ Nie
5. Chciałabym/chciałbym w większym stopniu uczestniczyć w podejmowaniu decyzji dotyczących mojego leczenia
   - ☐ Tak
   - ☐ Nie
6. Chciałabym/chciałbym, aby lekarze i pielęgniarki przekazywali mi informacje bardziej zrozumiałym językiem/w bardziej zrozumiały sposób
   - ☐ Tak
   - ☐ Nie
7. Chciałabym/chciałbym, aby lekarze byli ze mną bardziej szczerzy
   - ☐ Tak
   - ☐ Nie
8. Potrzebuję bliższego kontaktu z lekarzem
   - ☐ Tak
   - ☐ Nie
9. Chciałabym/chciałbym, aby moje objawy (np. ból, nudności, bezsenność) były lepiej łagodzone
   - ☐ Tak
   - ☐ Nie
10. Potrzebuję więcej pomocy przy czynnościach takich jak jedzenie, ubieranie się, korzystanie z toalety
    - ☐ Tak
    - ☐ Nie
11. Potrzebuję większego poszanowania mojej intymności/prywatności
    - ☐ Tak
    - ☐ Nie
12. Chciałabym/chciałbym, aby pielęgniarki okazywały mi więcej uwagi/zainteresowania
    - ☐ Tak
    - ☐ Nie
13. Chciałabym/chciałbym, aby lekarze umieli rozpraszać moje wątpliwości

- Tak
- Nie

14. Chciałabym/chciałbym, aby w szpitalu były lepsze warunki (np. stan łazienek, jakość posiłków)
    - Tak
    - Nie
15. Potrzebuję więcej informacji dotyczących należnych mi świadczeń socjalnych związanych z moją chorobą
    - Tak
    - Nie
16. Potrzebuję pomocy finansowej
    - Tak
    - Nie
17. Chciałabym/chciałbym mieć możliwość spotkania/rozmowy z psychologiem
    - Tak
    - Nie
18. Chciałabym/chciałbym mieć możliwość spotkania/rozmowy z księdzem
    - Tak
    - Nie
19. Chciałabym/chciałbym mieć możliwość spotkania/rozmowy z osobami, które mają taką samą chorobę
    - Tak
    - Nie
20. Chciałabym/chciałbym, aby moi bliscy bardziej mnie wspierali
    - Tak
    - Nie
21. Chciałabym/chciałbym czuć się bardziej potrzebna/y mojej rodzinie
    - Tak
    - Nie
22. Chciałabym/chciałbym czuć się mniej samotna/y
    - Tak
    - Nie
23. Chciałabym/chciałbym, aby ludzie okazywali mi mniej współczucia/litości
    - Tak
    - Nie

METRYCZKA Nr ………

1. Płeć:
- kobieta
- mężczyzna

2. Wiek: …………lat

3. Wykształcenie:
- podstawowe
- średnie/zawodowe
- wyższe

4. Miejsce zamieszkania
- miasto, jakie ….……………….
- wieś

5. Aktywność zawodowa
- uczeń/student
- nadal pracuję zawodowo
- w chwili obecnej L4 lub zasiłek rehabilitacyjny
- bezrobotny
- emeryt
- rencista

6. Stan cywilny
- mężatka/żonaty lub w stałym związku
- mój związek zakończył się w trakcie lub w związku z chorobą
- panna/kawaler
- rozwiedziona/rozwiedziony
- wdowa/wdowiec

7. Mieszkam
- tylko ze współmałżonkiem lub partnerem/partnerką
- ze współmałżonkiem lub partnerem/partnerką i z dzieckiem/dziećmi
- tylko z dzieckiem/dziećmi
- z innym członkiem rodziny
- samotnie

8. Czy ktoś z Pani/Pana najbliższej rodziny lub przyjaciół jest lekarzem?
   - tak
   - nie

9. Rozpoznanie choroby:……………………………………………

10. Przybliżona data rozpoznania choroby:…………………………
(miesiąc/rok)
