# Supplementary material for: Validation of the Standardized Needs Evaluation Questionnaire in Polish Cancer Patients
Source: Cancers (Basel). 2024 Apr 9;16(8):1451. doi: 10.3390/cancers16081451 (PMC11048258; doi:10.3390/cancers16081451)
Supplement: Supplementary file 1 [file cancers-16-01451-s001.zip › cancers-2919324-supplementary/Supplementary material/Suppl. 2.pdf]

Suppl. 2. English version of Needs Evaluation Questionnaire

QUESTIONNAIRE NUMBER……………………..

1. I need more information about my diagnosis
   - ☐ Yes
   - ☐ No
2. I need more information about my future condition
   - ☐ Yes
   - ☐ No
3. I need more information about the exams I am undergoing
   - ☐ Yes
   - ☐ No
4. I need more explanations of treatments
   - ☐ Yes
   - ☐ No
5. I need to be more involved in the therapeutic choices
   - ☐ Yes
   - ☐ No
6. I need clinicians and nurses to give me more comprehensible information
   - ☐ Yes
   - ☐ No
7. I need clinicians to be more sincere with me
   - ☐ Yes
   - ☐ No
8. I need to have a better dialogue with Clinicians
   - ☐ Yes
   - ☐ No
9. I need my symptoms (pain, nausea, insomnia, etc.) to be better controlled
   - ☐ Yes
   - ☐ No
10. I need more help with eating, dressing, and going to the bathroom
    - ☐ Yes

- No

11. I need better respect for my intimacy
   - Yes
   - No
12. I need better attention from nurses
   - Yes
   - No
13. I need to be more reassured by the clinicians
   - Yes
   - No
14. I need better services from the hospital (bathrooms, meals, cleaning)
   - Yes
   - No
15. I need to have more economic insurance information (tickets, invalidity, etc..) In relation to my illness
   - Yes
   - No
16. I need economic help
   - Yes
   - No
17. I need to speak with a psychologist
   - Yes
   - No
18. I need to speak with a spiritual advisor
   - Yes
   - No
19. I need to speak with people who have this same experience
   - Yes
   - No
20. I need to be more reassured by my relatives
   - Yes
   - No
21. I need to feel more useful within my family

- Yes
- No

22. I need to feel less abandoned
    - Yes
    - No
23. I need to receive less commiseration from other people
    - Yes
    - No

DEMOGRAPHIC AND CLINICAL DATA

1. Gender:
- ☐ female
- ☐ male

2. Age: …………years

3. Education:
- ☐ primary
- ☐ secondary
- ☐ high

- ☐ Place of residence
- ☐ city, ……………….
- ☐ village

4. Professional activity
- ☐ student
- ☐ active
- ☐ active, but actually sick leave
- ☐ unemployed
- ☐ pensioner
- ☐ disability pensioner

6. Marital status
- ☐ married or in a stable informal relationship
- ☐ relationship broken during disease or in relation to disease
- ☐ single
- ☐ divorced
- ☐ widow/widower

7. Living with:
- ☐ partner
- ☐ partner and child/children
- ☐ child/children
- ☐ another family member
- ☐ alone

8. Do you have a medical doctor as a close family or friend?
   - ☐ yes
   - ☐ no

9. Cancer:……………………………………………………..

10. Approximate date of cancer diagnosis:…………………………
(month/year)
