# Supplementary material for: Validation of the Standardized Needs Evaluation Questionnaire in Polish Cancer Patients
Source: Cancers (Basel). 2024 Apr 9;16(8):1451. doi: 10.3390/cancers16081451 (PMC11048258; doi:10.3390/cancers16081451)
Supplement: Supplementary file 1 [file cancers-16-01451-s001.zip › cancers-2919324-supplementary/Supplementary material/Suppl. 3.pdf]

Suppl. 3. Polish version of Validation Procedure Questionnaire

WALIDACJA Nr ……….

I. Czas wypełniania ankiety

1. właściwej ankiety: ……….. minut
2. metryczki: …..…………….. minut

II. Zrozumiałość, akceptowalność ankiety

1. Czy forma ankiety jest według Pani/Pana dobra?

☐ Tak

☐ Nie

2. Czy wielkość liter jest według Pani/Pana wystarczająco duża?

☐ Tak

☐ Nie

3. Czy sądzi Pani/Pan, że ankieta jest odpowiednio długa?

☐ Tak

☐ Nie – powinna być krótsza

☐ Nie – powinna być dłuższa

4. Czy pytania są według Pani/Pana generalnie zrozumiałe?

☐ Tak

☐ Nie

5. Czy były pytania, na które trudno było Pani/Panu odpowiedzieć jednoznacznie?

☐ Tak

Które? Nr Pytania………..

☐ Nie

6. Czy były pytania, na które Pani/Pan nie mieli ochoty odpowiadać?

☐ Tak

Które? Nr Pytania………..

☐ Nie

7. Czy jest coś jeszcze, o czym Pani/Pan chciałby powiedzieć odnośnie swoich potrzeb?

☐ Tak

Co takiego?…………………………………………………………………………...

☐ Nie

8. Czy myśli/sądzi Pani/Pan, że wypełnienie takiej ankiety może pomóc w lepszym kontakcie z lekarzem/pielęgniarką/innym personelem?

☐ Tak

- ☐ Nie

9. Czy dzięki tej ankiecie zauważyła Pani/Pan coś ważnego, na co wcześniej nie zwracała Pani/Pan uwagi?

- ☐ Tak

  Co takiego?...

  Ewentualnie Nr Pytania ………..
- ☐ Nie
