# Supplementary material for: Validation of the Standardized Needs Evaluation Questionnaire in Polish Cancer Patients
Source: Cancers (Basel). 2024 Apr 9;16(8):1451. doi: 10.3390/cancers16081451 (PMC11048258; doi:10.3390/cancers16081451)
Supplement: Supplementary file 1 [file cancers-16-01451-s001.zip › cancers-2919324-supplementary/Supplementary material/Suppl. 4.pdf]

Suppl. 4. English version of Validation Procedure Questionnaire

VALIDATION No ……….

I. Time of completion the questionnaire

1. main questionnaire: ……….. minutes
2. demographics: ….……….. minutes

II. Comprehensibility and acceptability of the questionnaire

1. Is the form of the questionnaire good in your opinion?

☐ Yes

☐ No

2. Is the font size big enough in your opinion?

☐ Yes

☐ No

3. Do you think that the questionnaire is sufficiently long?

☐ Yes

☐ No – should be shorter

☐ No – should be longer

4. Are the questions generally understandable in your opinion?

☐ Yes

☐ No

5. Are any questions difficult for you to answer clearly?

☐ Yes

Which? Number of question ………..

☐ No

6. Are any questions you do not want to answer?

☐ Yes

Which? Number of question ………..

☐ No

7. Is there anything else you would like to tell about your needs?

☐ Yes

What?………………………………………………………………………...

☐ No

8. Do you think that completing this questionnaire may facilitate better contact with the doctor / nurse / other staff?

- ☐ Yes
- ☐ No

9. Did you identify any important needs, which you did not recognize before the questionnaire?

- ☐ Yes

  What?...

  Number of question ………..
- ☐ No
