# Supplementary material for: Validation of the Standardized Needs Evaluation Questionnaire in Polish Cancer Patients
Source: Cancers (Basel). 2024 Apr 9;16(8):1451. doi: 10.3390/cancers16081451 (PMC11048258; doi:10.3390/cancers16081451)
Supplement: Supplementary file 1 [file cancers-16-01451-s001.zip › cancers-2919324-supplementary/Supplementary material/Suppl. 6.pdf]

Suppl. 6. English version of consent form

CONSENT FORM

I give my informed consent to participate in the study entitled: "Validation of the standardized NEQ (Needs Evaluation Questionnaire) in Polish cancer patients".

I give my consent to use the information obtained using the Needs Evaluation Questionnaire for anonymous analysis and publication in form a manuscript.

I know that participation in the study is anonymous. I have been informed that my personal information will not be disclosed in accordance with the Act of May 10, 2018 on the Protection of Personal Data.

....	....
Date	Sign of participant
....	....
Date	Sign of person providing information
